# Removal of HCl from a gas phase by MgO under atmospheric conditions

**DOI:** 10.1080/14686996.2025.2454215

**Published:** 2025-01-31

**Authors:** Michiko Kitagawa, Hiromi Matsuhashi, Masanori Kidera, Kazuya Takahashi, Takahiro Kondo

**Affiliations:** a Graduate School of Science and Technology, University of Tsukuba, Tsukuba, Ibaraki, Japan; b Nishina Center for Accelerator-Based Science, RIKEN, Wako, Saitama, Japan; c Department of Science, Hokkaido University of Education, Hakodate, Hokkaido, Japan; d Department of Materials Science, Institute of Pure and Applied Sciences, University of Tsukuba, Ibaraki, Japan; e Tsukuba Research Center for Energy Materials Science, Institute of Pure and Applied Sciences and R&D Center for Zero CO_2_ Emission with Functional Materials, University of Tsukuba, Tsukuba, Ibaraki, Japan; f Advanced Institute for Materials Research, Tohoku University, Sendai, Miyagi, Japan

**Keywords:** MgO, remove HCl, surface area, active site, atmospheric condition, base, catalyst

## Abstract

Ensuring the safety of researchers by protecting them from exposure to toxic gases in laboratories is of paramount importance. This study investigated the effectiveness of using high-surface-area MgO to remove HCl under atmospheric conditions. Two types of MgO were synthesized through the thermal decomposition 1-1-1, Tennodai, Tsukuba, of Mg(OH)_2_ and MgC_2_O_4_·2 H_2_O. HCl diluted with air passed through both MgO samples, and the amounts of HCl removed and morphological changes in the samples were compared. No significant differences in surface area or crystallinity were observed with the decomposition temperatures. X-ray diffraction analysis showed that the sample prepared from MgC_2_O_4_·2 H_2_O reacted with HCl immediately upon introducing HCl gas. In contrast, the sample obtained from Mg(OH)_2_ exhibited only MgO peaks, even 30 min after the introduction of HCl gas. Microscopic analysis revealed that the samples derived from Mg(OH)_2_ showed no significant changes in shape after the reaction, whereas the MgO prepared from MgC_2_O_4_·2 H_2_O exhibited substantial changes in overall shape. No correlation was observed between the surface area and the amount of HCl removed. When MgO is prepared from MgC_2_O_4_·2 H_2_O, the reaction occurs in the bulk material, whereas when MgO is prepared from Mg(OH)_2_, the reaction hardly progresses after HCl adsorbs onto the MgO surface. The order of magnitude of HCl removal was consistent with the base catalytic activity of the decomposition of diacetone alcohol to acetone. These results suggest that, compared with MgO obtained from Mg(OH)_2_, MgO derived from MgC_2_O_4_·2 H_2_O generates more active sites, resulting in the reaction with HCl from surface to progress into bulk.

## IMPACT STATEMENT

This study revealed that MgO derived from MgC_2_O_4_·2H_2_O generates more active sites that initiate the reaction with HCl under atmospheric conditions compared with MgO obtained from Mg(OH)_2_

## GRAPHICAL ABSTRACT

**Figure:**
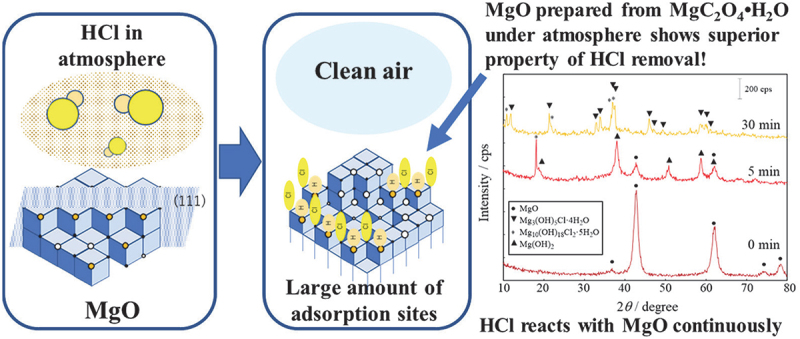

## 1. Introduction

Hydrogen chloride is a toxic gas produced during the synthesis and decomposition of chlorine-containing chemical warfare agents (CWAs), such as mustard gas, phosgene and benzyl chloride. CWAs are typically handled in confined spaces, such as laboratories. To ensure safety, decontamination methods should be tailored based on the location and context of use, such as development laboratories, manufacturing sites, storage facilities, and decomposition sites [1]. Consequently, extensive research has been conducted on the detection and decontamination of CWAs, such as the use of nanosized MgO for CWA decomposition [2–4].

In the 1990s, CWAs were used in terrorist attacks in Japan, with sodium carbonate solution primarily employed for decontamination. In confined spaces, such as laboratories, MgO is considered an effective agent for decontaminating CWAs. Because MgO has a low molecular weight, the amount removed per unit weight is substantial. Notably, Klabunde et al. investigated the adsorption of various acid gases such as SO_2_, CO_2_, and HCl using high-surface area nanosized MgO particles [5]. Stark et. al found that a substantial amount of HCl could be adsorbed onto the surface of MgO [6]. However, in their adsorption tests of various acidic gases, the gases were introduced at the desired pressure under high-vacuum conditions. Therefore, investigating the atmospheric adsorption capacity of MgO is crucial for its application in laboratories and manufacturing sites.

MgO is a well-known solid-base catalyst. The active solid base of MgO is typically prepared through the thermal decomposition of hydroxide or basic magnesium carbonate at high temperatures. MgO exhibits high catalytic activity for the hydrogenation of alkenes, anion-radical formation, and several base-catalyzed reactions [7]. However, the optimal thermal treatment temperatures for these reactions vary [7]. Researchers have focused on developing different preparation methods to increase the surface area and activity of this base catalyst, which features a simple structure and unique characteristics [8,9]. In general, the preparation of MgO as a base catalyst is simple. Precursors such as hydroxide, basic carbonate, or oxalate are heated in air or high vacuum at 673 K or higher for a few hours. However, the adsorption of CO_2_ and/or H_2_O from the air can readily deactivate the active sites on the surface of MgO [10]. Consequently, various studies have been conducted under vacuum conditions to prevent the poisoning of active sites by CO_2_ and/or H_2_O. This poisoning phenomenon has led to the inconvenient handling of MgO and made its practical use challenging. However, in our recent study, Mg(OH)_2_ was thermally decomposed in the atmosphere at low temperatures for a short duration to obtain high-surface-area MgO suitable for retro – aldol reactions [11]. Mg(OH)_2_, which is the most commonly used starting material, was employed to investigate how its decomposition temperature and heating time affect the base catalytic activity of MgO. The optimal decomposition conditions were determined to be heating at 673 K for 20 min. Notably, this set of conditions is considerably lower in temperature and shorter in duration than conventional conditions. Although all treatments, including decomposition, were conducted under atmospheric conditions, the MgO prepared by this method exhibited sufficient base catalytic activity.

The effects of various starting materials have also been studied. For example, a previous study compared the basic activities of MgO samples prepared through the thermal decomposition of hydroxide, carbonate, and oxalate, revealing that magnesium oxalate was the most suitable starting material [12]. Although the decomposition of magnesium oxalate and all experiments were performed under atmospheric conditions, MgO exhibited a large surface area and high activity.

Itoh et al. reported that autoclave prepared MgO (denoted as AP-MgO), with a high surface area exceeding 400 m^2^/g, could be prepared using an autoclave hypercritical drying procedure [13]. Furthermore, Stark et al. found that AP-MgO could effectively remove HCl [6], and a high HCl removal rate under high-vacuum condition was achieved when the MgO possessed a large surface area, irrespective of the synthesis method.

In this study, we prepared MgO with a large surface area and high activity under atmospheric conditions using different starting materials. One method involved the short-term thermal decomposition of hydroxide, while the other employed the thermal decomposition of magnesium oxalate. The removal of HCl, which is a representative acidic gas, was examined to understand the different reactivities between MgO samples prepared under different conditions. These variations in reactivity with HCl were analyzed and discussed using X-ray diffraction (XRD), ion chromatography, scanning electron microscopy (SEM), surface area values, and base catalytic activity for decomposing diacetone alcohol to acetone.

## 2. Experimental

### 2.1. Materials

In this study, MgO samples were prepared by thermally decomposing MgC_2_O_4_·2 H_2_O or Mg(OH)_2_ to compare their differences in HCL removal. Furthermore, MgO was prepared from Mg(OH)_2_ by two different methods to compare the conventional and new preparation methods. MgC_2_O_4_·2 H_2_O was used as received (FUJIFILM Wako Pure Chemical Corporation; Osaka, Japan; Lot. SAK1542). Mg(OH)_2_ was prepared as follows: Pure MgO (Merck KGaA; Darmstadt, Germany; Lot. TA1760165) was placed in a beaker and heated in distilled water for 1 h to obtain Mg(OH)_2_ [14]. To remove the water from the suspension and obtain Mg(OH)_2_, the Mg(OH)_2_ in the aqueous solution was then collected via filtration and dried at 373 K for 12 h. Filtration was performed using Advantec filter paper No. 2 (Toyo Roshi Kaisya, Ltd.; Tokyo, Japan).

### 2.2. Synthesis of MgO using MgC_2_O_4_·2 H_2_O

MgO was prepared from MgC_2_O_4_·2 H_2_O via thermal decomposition in a muffle furnace (NITTO KAGAKU Co., LTD.; Nagoya, JAPAN; MODEL NHK-120 h) at 773–1173 K for 3 h.

### 2.3. Synthesis of MgO using Mg(OH)_2_

The thermal decomposition of Mg(OH)_2_ into MgO was performed in a muffle furnace. The conventional procedure for MgO preparation was as follows: Mg(OH)_2_ was placed in a furnace at approximately 298 K. The furnace temperature was increased to 873 K over 40 min and maintained for 3 h. In contrast, the MgO prepared through short-time heating was conducted as follows: Mg(OH)_2_ was placed in a furnace maintained at 673–1073 K, and the sample was removed from the furnace after 20 min. All samples were placed in the gas flow system immediately after cooling to approximately 298 K under atmospheric conditions.

### 2.4. Characterization

The surface area of MgO was measured using the Brunauer – Emmett – Teller (BET) method (MicrotracBEL; Osaka, Japan; BELSORP MINIX). Prior to the measurement, the samples were treated at 573 K for 2 h under vacuum to remove adsorbed species from the surface. Nitrogen adsorption/desorption isotherms (*V*_a_ vs *P*/*P*_0_) were measured at 77 K. Pore volume was calculated by the Barrett-Joyner-Halenda (BJH) method.

XRD patterns of the as-prepared MgO samples were measured using the RIGAKU Ultimate IV instrument (Tokyo, Japan) with Cu Kα_1_ radiation (1.5406 Å). The diffraction patterns of the MgO samples after passing the HCl-mixed dry air for 5 and 30 min were obtained using a RIGAKU Ultima+ instrument (Tokyo, Japan) at a wavelength of 1.54184 Å (average of a 2:1 mixture of Cu Kα_1_ and Kα_2_). The crystallite size of MgO was calculated from the XRD results using the Scherrer equation *D* = *K λ*/*β* cos *θ*, (*K*: dimensionless shape factor, typically considered as ~ 0.9, *D*: crystal size, *β*: diffraction peak half-width, and *λ*: wavelength of incident X-rays). The samples before and after HCl removal were observed using SEM (Thermo Fisher Scientific; Tokyo, Japan; Quattro S) at an accelerating voltage of 20 kV.

### 2.5. Measurement of the HCl removal property

Hydrogen chloride removal from the gas phase was conducted at approximately 298 K. After placing MgO in the reactor, commercially available HCl gas diluted with dry air was introduced. Next, 5.15% HCl gas was passed through the reactor at a flow rate of 10 mL/min, and dry air was introduced as a carrier gas at a flow rate of 20 mL/min under atmospheric conditions. The outflow gases were analyzed using a mass spectrometer (PFEIFFER VACUUM; New Hampshire, US; PrismaPlus). The amount of removed HCl was calculated by subtracting the blank integral from the integral area of the current plotted against time. Specifically, the time course of HCl (m/z = 36) and H_2_O (m/z = 18) in the gas phase was observed when HCl mixed with dry air passed through MgO prepared from MgC_2_O_4_·2 H_2_O and Mg(OH)_2_. The amount of HCl removed from each sample was calculated after the factor correction by considering the mass spectrum sensitivity at each measurement. In this work, the time course of water observed at m/z = 18 could not be evaluated in absolute terms because the passing gas was a mixture of dry air and HCl, which did not contain water to serve as a standard. In addition, water was readily absorbed by the walls of the pipes through which the gas passed, and the amount of water absorbed could vary depending on the laboratory environment at any given time. In all samples, the amount of HCl decreased rapidly immediately after the gas was introduced.

### 2.6. Measurement of the chloride concentrations in the sample

The chloride concentrations of the samples were analyzed using ion chromatography (Thermo Fisher Scientific; Tokyo, Japan; ICS-2000 or ICS-3000) or energy-dispersive X-ray spectroscopy (EDS) (RIGAKU; Tokyo, Japan; EDXL300).

## 3. Result and discussion

### 3.1. Nitrogen adsorption/desorption isotherms and surface area

**Figure 1.**
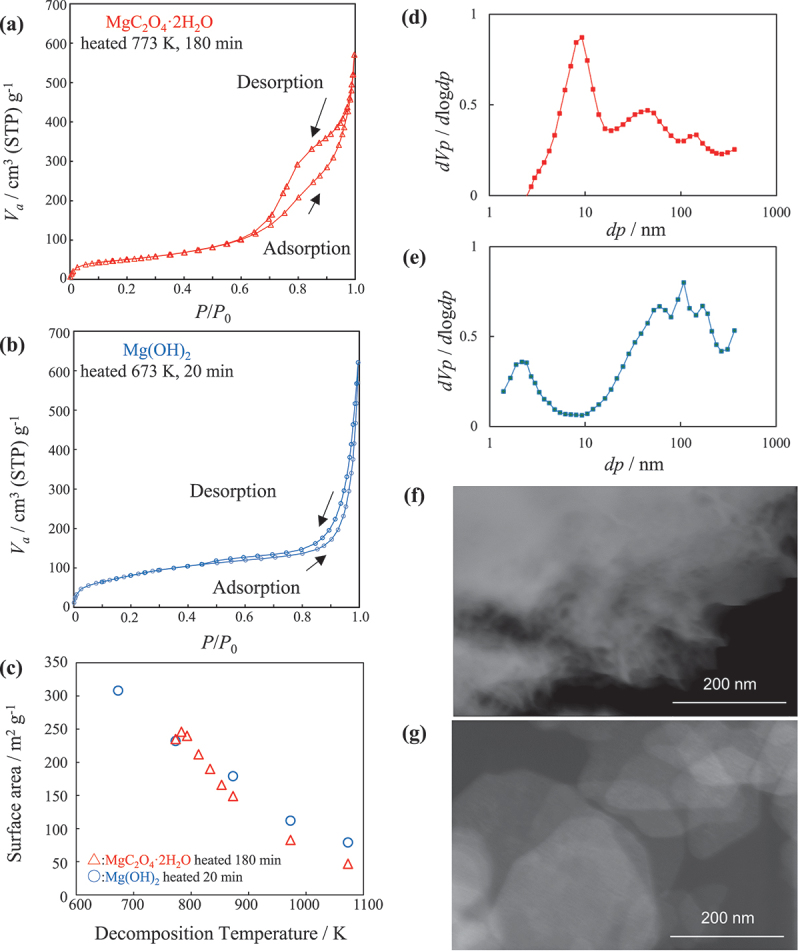
Adsorption/desorption isotherms and surface area of MgO. Nitrogen adsorption/desorption isotherms at 77 K for (a) the MgC_2_O_4·_2 H_2_O sample heated at 773 K for 180 min and (b) the Mg(OH)_2_ sample heated at 673 K for 20 min. (c) Surface area, calculated using the BET method, versus the thermal decomposition temperature of MgO obtained from MgC_2_O_4·_2 H_2_O and Mg(OH)_2_. Pore size distribution calculated by the BJH method from the adsorption isotherms of (d) MgO prepared from MgC_2_O_4·_2 H_2_O and (e) MgO prepared from Mg(OH)_2_. SEM image of (f) MgO prepared by heating MgC_2_O_4·_2 H_2_O at 773 K for 180 min and (g) MgO prepared by heating Mg(OH)_2_ at 673 K for 20 min.

The adsorption and desorption isotherms of MgC_2_O_4_·2 H_2_O and Mg(OH)_2_ thermally decomposed at 773 K for 3 h and Mg(OH)_2_ thermally decomposed at 673 K for 20 min are shown in Figure 1a,b, respectively. Hysteresis can be observed in the isotherms of MgO prepared from MgC_2_O_4_·2 H_2_O (Figure 1a). The pore volumes of the MgO samples prepared from MgC_2_O_4_·2 H_2_O and Mg(OH)_2_ were 0.84 cm^3^ g^−1^and 0.92 cm^3^ g^−1^, respectively. The MgO prepared from MgC_2_O_4_·2 H_2_O showed pore sizes of approximately 10 nm, which could be classified as mesopores (Figure 1d). In addition, a peak at approximately 100 nm was observed in the MgO prepared from Mg(OH)_2_ (Figure 1e). Mg(OH)_2_ has a brucite structure. Upon heating, the layered Mg(OH)_2_ was dehydrated and converted to MgO [15], i.e. MgO retained the morphology of the original Mg(OH)_2_ (Figure 1g). Therefore, the pore distribution and pore volume in the MgO prepared from Mg(OH)_2_ are considered to be affected by the adsorption between layers rather than composing 100 nm pores. Figure 1c shows the estimated surface area of MgO by BET analysis for the samples prepared from MgC_2_O_4_·2 H_2_O and Mg(OH)_2_ as a function of thermal decomposition temperature. The thermal decomposition temperature of MgC_2_O_4_·2 H_2_O is 756 K; therefore, the thermal decomposition temperature of MgO derived from this precursor was set to 773–1073 K. When both raw materials underwent thermal decomposition, the surface area decreased monotonically with increasing temperature. Moreover, these raw materials showed no significant difference in surface area at the same thermal decomposition temperature.

### 3.2. XRD analysis

**Figure 2.**
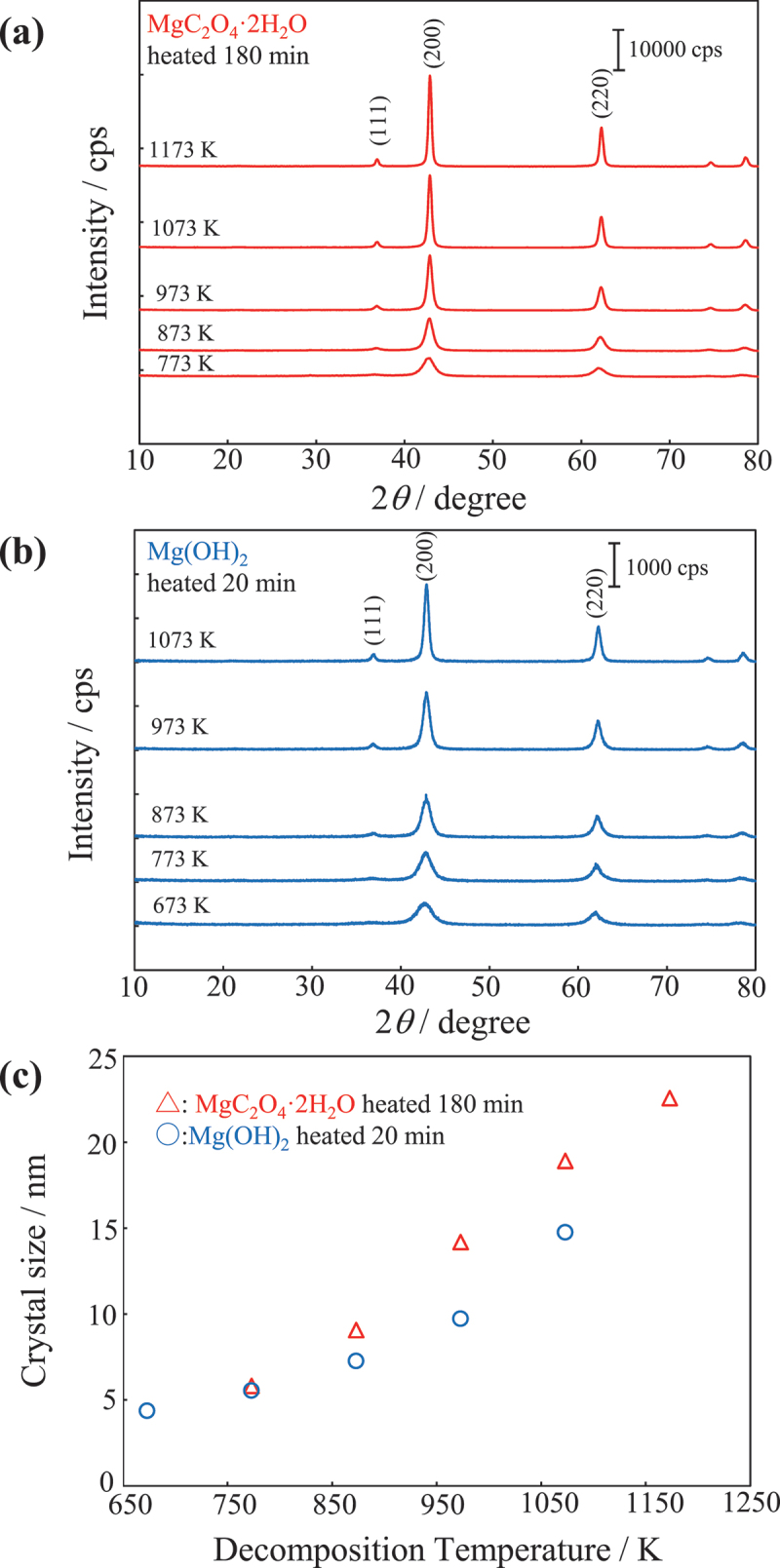
XRD profiles and crystal sizes of MgO. XRD profiles of MgO prepared by thermally decomposing (a) MgC_2_O_4_·2 H_2_O at 773–1173 K for 180 min [11] and (b) Mg(OH)_2_ at 773–1173 K for 20 min. (c) Crystallite size of the MgO versus the thermal decomposition temperature. The crystal size was estimated based on the peak width of the (200) plane in the XRD pattern using the Scherrer equation.

The XRD results for MgC_2_O_4_·2 H_2_O and Mg(OH)_2_ at each thermal decomposition temperature are shown in Figure 2a,b, respectively [12]. A set of MgO peaks can be observed at all decomposition temperatures, indicating that MgO was formed from both raw materials across the entire temperature range [16]. For both raw materials, the peaks sharpen as the thermal decomposition temperature increases. Figure 2c shows the crystallite size calculated from the MgO (200) plane peak of the XRD data using the Scherrer equation. Since MgO has a face-centered cubic structure, the peak of the (100) plane cannot be observed due to extinction rule. Therefore, the crystallite size was calculated from the peak of the (200) plane at 42.9° as shown in Figure 2c. The crystallite size increased with increasing thermal decomposition temperature for MgO prepared from either MgC_2_O_4_·2 H_2_O or Mg(OH)_2_. The surface area and crystallinity of MgO prepared from these raw materials exhibit similar trends with respect to thermal decomposition temperature.

### 3.3. HCl removal from vapor phase

**Figure 3.**
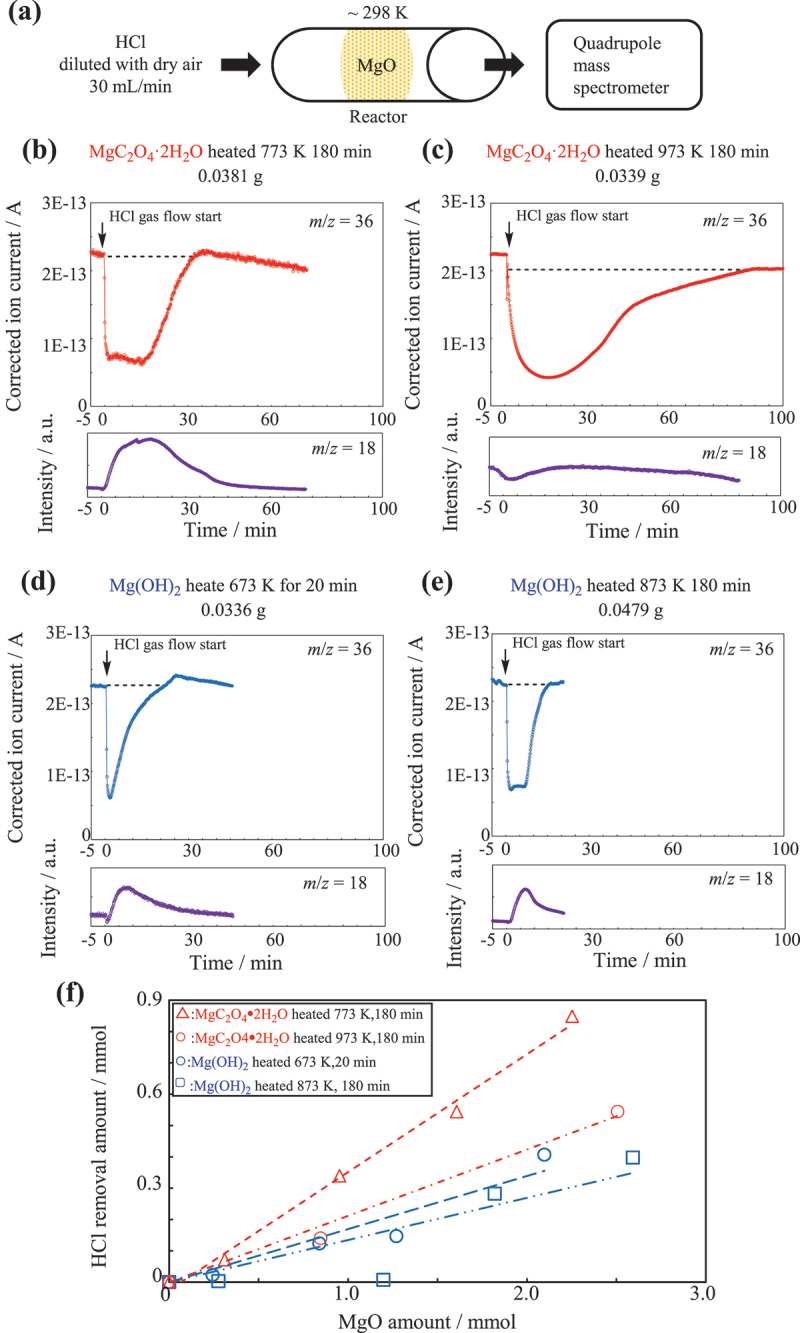
Measurement of the HCl removal capacity. (a) Schematic of a fixed-bed HCl gas distribution device. Time course of HCl and H_2_O content in effluent gas passed through MgO prepared by thermally decomposing (b) MgC_2_O_4_·2 H_2_O at 773 K for 180 min, (c) MgC_2_O_4_·2 H_2_O at 973 K for 180 min, (d) Mg(OH)_2_ at 673 K for 20 min, and (e) Mg(OH)_2_ at 873 K for 180 min. (f) Relationship between the amount of HCl removed from the gas phase and the sample amount. The amount of removed HCl was calculated based on the integrated area of the HCl time course measured using quadrupole mass spectrometry.

Previous reports have studied the HCl adsorption using nano-sized MgO particles [6], as well as the chlorination of MgO with HCl gas [17]. However, in these studies, the experimental conditions for HCl gas adsorption were under vacuum and at high temperatures, respectively. The objective of this study is to ensure a safe research environment by using MgO for HCl removal. Therefore, all work, such as the preparation of MgO and HCl removal tests, was carried out under atmospheric conditions. HCl mixed with dry air passed through the sample placed in a reaction tube, and the outlet gas was analyzed using quadrupole mass spectrometry (Figure 3a). As shown in Figure 3b, for MgO prepared from MgC_2_O_4_·2 H_2_O by thermal decomposition at 773 K, the amount of HCl decreased for several minutes, before rapidly returning to its original amount. Concurrently, the water content increased. Figure 3c shows that the amount of HCl decreased over a long term for the MgO sample prepared by thermally decomposing MgC_2_O_4_·2 H_2_O at 973 K. The water content also increased slightly during this period. In contrast, for the sample using MgO thermally decomposed from Mg(OH)_2_ at 673 K, the amount of HCl decreased rapidly after the gas was introduced, but subsequently returned gradually to its original amount (Figure 3d). The water content also increased slightly. For MgO obtained through the thermal decomposition of Mg(OH)_2_ at 873 K for 3 h, HCl removal and water content increase occurred rapidly (Figure 3e).

In the HCl removal test using MgO prepared through short-term decomposition (Figure 3d), removal was presumably achieved via surface adsorption because of the very short response time. In contrast, for the cases shown in Figures 3b,c,e, where decomposition was performed over an extended period, two types of reactions may have occurred: adsorption on the surface and a reaction into the bulk. Because water production was observed in all the samples, the following reaction likely occurred: MgO + 2HCl → MgCl_2_ + H_2_O. Figure 3f shows a plot of the amount of HCl removed from the gas phase versus the amount of MgO sample. In all samples, the amount of HCl removed increased proportionally to the amount of MgO. The slope of this plot was calculated as the reactivity (Table 1). In addition, the Cl^−^ concentration in the MgO samples after HCl removal was measured using ion chromatography, as shown in Table 1, along with the surface area and base catalytic activity. The MgO obtained by thermally decomposing MgC_2_O_4_·2 H_2_O at 773 K for 3 h, exhibited the highest HCl removal. This MgO sample exhibited a comparable or greater capacity for HCl removal when compared with the results reported by Stark et al. [6], where nanoscale and microscale MgO particles were used for adsorption of acidic gases, including HCl. In their experiments, the MgO was exposed to 100 Torr of HCl gas, and the amount of gas adsorbed was determined based on the change in the weight of the MgO. A key advantage of our study is that all operations, including preparation of MgO and HCl removal, were carried out under atmospheric conditions. The order of magnitude of HCl removed was consistent with the base catalytic activity for decomposing diacetone alcohol to acetone [12]. It has been reported that the three-coordinated O^2-^ in MgO is the active site of the retro-aldol reaction, and the number of this active site decreases when thermal decomposition of Mg(OH)_2_ is conducted at high temperatures for a long period of time [11]. Therefore, the same three-coordinate O^2-^ is considered as active site for HCl removal (e.g. the adsorption site of HCl gas) as in the case for the retro-aldol reaction. The Cl^−^ concentrations in the samples after the HCl removal test exhibited a trend similar to that of the reactivity. When comparing samples obtained by thermally decomposing Mg(OH)_2_ at 873 K for 3 h and MgC_2_O_4_·2 H_2_O at 973 K for 3 h, both of which had similar surface areas, the sample derived from MgC_2_O_4_·2 H_2_O showed a higher Cl^−^ concentration after the test. This value is nearly identical to that of the sample prepared from Mg(OH)_2_ at 673 K for 20 min, which had over three times the surface area. Moreover, the MgO prepared from MgC_2_O_4_·2 H_2_O exhibited approximately twice the HCl removal capacity as the MgO prepared by heating Mg(OH)_2_ at 673 K for 20 min, despite having a significantly smaller surface area. These results clearly indicate that there is no correlation between the surface area and the amount of HCl removed.

**Table 1.** Surface area, HCl removal capacity, and base catalytic activity of MgO prepared from different raw materials and conditions.

Material	Decomposition condition	Surface area/m^2^ g^−1^	Reactivity/mol_HCl_ mol^−1^_MgO_ (Amount of HCl removed from the air)	Cl^−^ Concentration /wt%	Base catalytic activity [10, 11]/mol g^−1^ h^−1^
MgC_2_O_4_·2 H_2_O	773 k, 180 min	235	0.36	17.3	2.4
MgC_2_O_4_·2 H_2_O	973 K, 180 min	83	0.21	14.6	-
Mg(OH)_2_	673 K, 20 min	308	0.17	14.5	0.58
Mg(OH)_2_	873 K, 180 min	80	0.13	11.8	0.14
Nanoscale MgO [6]	773 K, 180 min**	302	0.25*	-	-
Microscale MgO [6]	773 K, 180 min**	177	0.19*	-	-

The surface area was determined using the BET plot. Reactivity was calculated from the slope of the line in Figure 3f. The Cl^−^ concentration was measured using ion chromatography after dissolving the entire sample following the HCl removal test. The base catalytic activity was determined from the catalytic reaction rate in the retro – aldol reaction of diacetone alcohol.

*The amounts of HCl adsorbed at 100 Torr for 15 min reported in reference [6].

**Pretreatment conditions reported in reference [6].

**Figure 4.**
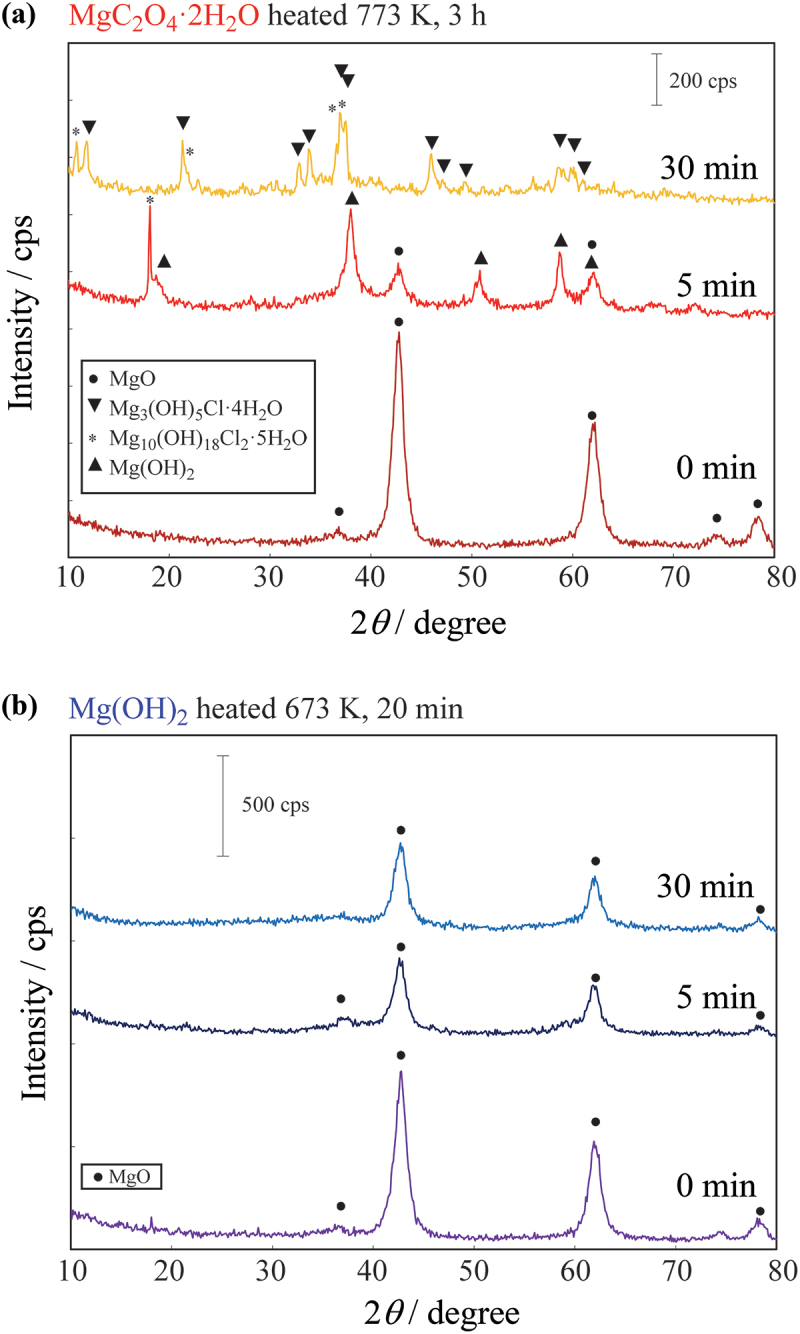
XRD profiles of MgO samples after passing HCl gas through for 0, 5, and 30 min. MgO prepared by thermally decomposing (a) MgC_2_O_4_·2 H_2_O at 773 K for 180 min and (b) Mg(OH)_2_ at 673 K for 20 min.

Figure 4 shows the XRD results obtained at 5 and 30 min after introducing HCl-mixed dry air through MgO prepared from MgC_2_O_4_·2 H_2_O and Mg(OH)_2_. For MgO prepared from MgC_2_O_4_·2 H_2_O, the XRD profile exhibits a set of Mg(OH)_2_ peaks at 5 min after the introduction of the HCl-mixed gas. Furthermore, at 30 min after the introduction of the gas, the profile shows a set of magnesium hydroxide chloride peaks. In contrast, the XRD patterns for MgO prepared from Mg(OH)_2_ show only the MgO peaks. These results suggest that in the sample prepared from MgC_2_O_4_·2 H_2_O, a reaction occurred immediately after the introduction of HCl gas, producing water. The XRD pattern of the sample obtained at 5 min after the gas was introduced indicates that the produced water reacted with MgO to form a hydroxide. In contrast, for MgO prepared from Mg(OH)_2_ at 673 K for 20 min either HCl did not react with the MgO after adsorption onto the surface, or the reaction occurred at a significantly slower rate compared with that of MgO prepared from MgC_2_O_4_·2 H_2_O. This is also strongly supported by the changes in the amount of HCl in the gas phase over time after the introduction of HCl (Figure 3). The amount of HCl in the gas that passed through MgO prepared from MgC_2_O_4_·2 H_2_O remained low for several minutes, however, for the MgO derived from Mg(OH)_2_, the amount immediately returned to its original level. These results indicate that for the MgO prepared from MgC_2_O_4_·2 H_2_O, HCl was adsorbed on the surface and subsequently reacted in the bulk, whereas for the MgO obtained from Mg(OH)_2_ (Figure 3d), HCl was only adsorbed on the surface.

**Figure 5.**
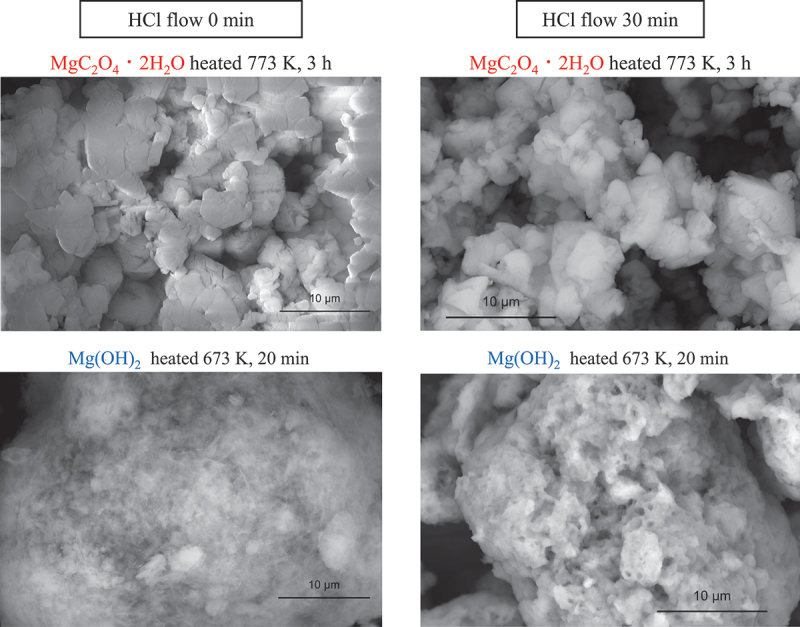
SEM images of MgO samples before and after passing HCl gas through them.

Figure 5 shows the SEM images of the samples obtained from MgC_2_O_4_·2 H_2_O and Mg(OH)_2_ raw materials before and after the HCl removal test. The shape of MgO varied considerably depending on the raw material used. After the HCl removal test, the MgO obtained from MgC_2_O_4_·2 H_2_O reacted as a whole and changed significantly in shape. The MgO prepared from Mg(OH)_2_ consisted of aggregates of fine hexagonal flakes derived from the shape of Mg(OH)_2_. After the HCl removal test, some shrinkage occurred; however, the shape did not change significantly. The marked change in the overall shape of the sample prepared from MgC_2_O_4_·2 H_2_O suggests that the reaction progressed throughout the entire sample. This aligns with the results in Figure 3, indicating that the reaction may have progressed throughout the bulk of the sample. In the case of MgO prepared from MgC_2_O_4_·2 H_2_O, the reaction may have occurred in the cracks or gaps between particles, as observed in the adsorption isotherm in Figure 1, and the reaction may have progressed into the interior of the sample.

**Figure 6.**
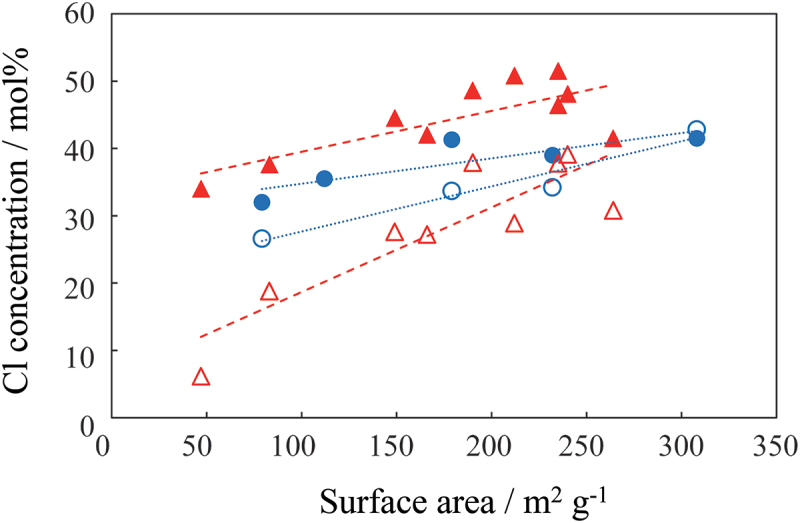
Cl concentration relative to Mg content in the samples after passing HCl gas through them. MgO obtained by thermally decomposing MgC_2_O_4_·2 H_2_O at 773 K for 180 min, with HCl passing for 30 (▲) and 15 (△) min. MgO obtained by thermally decomposing Mg(OH)_2_ at 673 K for 20 min, with HCl passing for 30 (●) and 15 (○) min.

Figure 6 shows the EDS measurements of the Cl concentration relative to the surface area of MgO prepared through the thermal decomposition of MgC_2_O_4_·2 H_2_O and Mg(OH)_2_ at 673–1173 K, following the passage of HCl for 15 and 30 min. In the samples prepared from MgC_2_O_4_·2 H_2_O, the Cl concentration increased significantly with increasing flow time. The smaller the surface area, the greater the difference in Cl concentration. When MgO prepared from Mg(OH)_2_ was used, the difference in the Cl concentration with respect to flow time was small. These results indicate that, for MgO prepared from Mg(OH)_2_, the HCl adsorbed on the surface reacted minimally with the sample over time. For MgO prepared from MgC_2_O_4_·2 H_2_O, the Cl concentration increased because the reaction continued throughout the sample. Compared with the case with an HCl gas flow time of 15 min, the MgO obtained by thermally decomposing Mg(OH)_2_ for 20 min at 673 K exhibited a higher Cl concentration, particularly in samples with a low surface area. This likely occurred because, for the MgO derived from Mg(OH)_2_, the adsorption reaction on the surface is rapid; however, after adsorption, the subsequent reaction progresses slowly.

### 3.4. Base catalytic activity and HCl removal capacity

**Figure 7.**
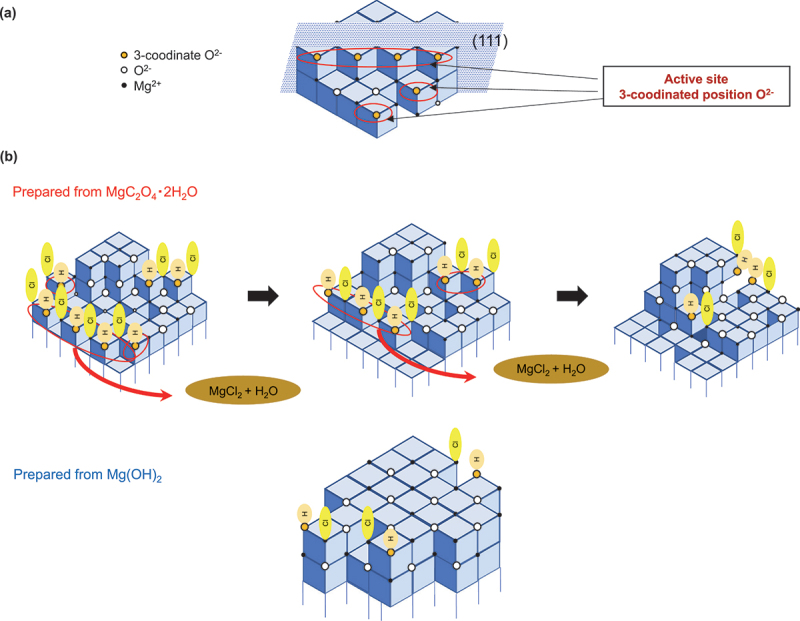
Active sites on MgO surfaces. (a) Three-coordinated oxygen atoms on the MgO surface. (b) Chemisorption of HCl onto the MgO surface prepared from different raw materials. The reaction progresses on the surface of MgO prepared from MgC_2_O_4_·2 H_2_O as new active sites emerge continuously.

The reaction between solid MgO and HCl begins with the chemisorption of HCl onto the MgO surface [18]. The adsorption sites of HCl, an acid gas, are thought to be the same as the base catalytic active sites on MgO, which are identified as three-coordinated O^2-^ ions (Figure 7a) [19,20]. As shown in Table 1, the trends in the base catalytic activity of MgO and its HCl removal capacity were consistent. It was found that a large amount of HCl was removed from gas phase by MgO prepared from MgC_2_O_4_·2 H_2_O, and that the reaction proceeded immediately after contact with HCl gas (Figure 4a and Table 1). From these results, it is assumed that there are abundant active sites on the surface of this sample, where HCl dissociates, adsorbs and reacts. Additionally, new active sites are generated by the desorption of magnesium chloride produced in the reaction, and the reaction proceeds in the bulk material (Figure 7b). In contrast, for the MgO obtained from Mg(OH)_2_, a very limited number of active sites are available as the initiation points, preventing the reaction from progressing.

## 4. Conclusion

In this study, MgO was obtained by thermally decomposing MgC_2_O_4_·2H_2_O or Mg(OH)_2_ under atmospheric conditions to maximize surface area, and its capacity to remove HCl from the atmosphere was investigated. The results indicate that the MgO obtained by thermally decomposing MgC_2_O_4_·2 H_2_O at 773 K for 3 h exhibited a significantly smaller surface area than the MgO obtained by thermally decomposing Mg(OH)_2_ at 673 K for 20 min; however, it removed over twice as much HCl. The changes in the samples over time owing to the reaction with HCl were examined using XRD and EDS, revealing that MgO prepared through the thermal decomposition of MgC_2_O_4_·2 H_2_O reacted immediately after the gas was introduced. This reaction likely continued throughout the bulk of the sample. The adsorption sites for HCl are assigned to be the same as the active sites of the base catalyst of the decomposition of diacetone alcohol to acetone. These results suggest that, compared with MgO obtained from the short-term thermal decomposition of Mg(OH)_2_, MgO derived from MgC_2_O_4_·2 H_2_O generates more active sites to react with HCl into bulk.
